# Early aggressive macrovascular disease and type 1 diabetes mellitus without chronic complications: a case report

**DOI:** 10.1186/1756-0500-6-222

**Published:** 2013-06-06

**Authors:** Alessandra Saldanha de Mattos Matheus, Marília Brito Gomes

**Affiliations:** 1 Diabetes Unit, State University of Rio de Janeiro, Rio de Janeiro, Brazil

**Keywords:** Diabetes mellitus, Type 1, Coronary artery disease, Macrovascular complications, Cardiovascular risk

## Abstract

**Background:**

Type 1 diabetes (T1DM) is considered to be one of the most significant risk factors for the development of coronary artery disease (CAD). However, the specific risk predictor models for T1DM are subject to many limitations.

**Case presentation:**

We report the case of a 42-year-old Caucasian woman presenting with T1DM for 26 years. During her chronic hyperglycemic evolution (mean of HbA1c > 3 percentage points above the superior limit) without microvascular complications, this patient presented with early and aggressive coronary artery disease, despite the lack of classical risk factors for CAD

**Conclusions:**

The rapidly progressive macrovascular disease observed in this case demonstrates the different degrees of aggressiveness and unpredictable clinical evolution observed in some cases. It also confirms the need for a multi-factorial, early and optimized clinical management regime.

## Background

Cardiovascular diseases are the main cause of morbidity and mortality among both type 1 and type 2 diabetes (DM) patients, affecting almost half of all diabetics. Its total prevalence has been estimated at about 55% of diabetic patients, as compared to 2-4% of the total population [1].

Type 1 diabetes (T1DM) is considered one of the most significant risk factors for the development of coronary artery diseases (CAD). However, it should be noted that, to date, the specific risk predictor models for T1DM are subject to many limitations [2]. The risk of CAD is expected to increase between two and four times [3], and DM is the third most important risk factor for this disease pathogenesis [4]. Consequently, diabetes also increases the risk of acute coronary syndromes (ACS), the incidence of which reaches the level of 20% within 7 years, as compared to an incidence of 3.5% among non-diabetics – an increase in incidence similar to individuals who had previously suffered an acute myocardial infarction (AMI) [3]. In Brazil, fasting glucose above 126 mg/dL corresponds to a three-fold increase in the risk for AMI [5]. It is also important to identify any of the well-determined specific risk factors for T1DM, such as nephropathy and autonomic neuropathy, as well as further subclinical atherosclerosis, which may already be affecting these patients at a late stage of disease progression [6]. When compared to non-diabetics, T1DM patients show more extensive lesions, a reduced ejection fraction of the left ventricle (LV), more cardiac events and a higher frequency of silent ischemia. They still present impairment of microcirculation and endothelial dysfunction that contributes to tissue perfusion disturbance [1,5]. Another intriguing fact is that T1DM reduces the differences between genders with respect to coronary calcification, with more significant effects on calcification seen in women relative to men [7].

The present case report describes the case of a female type 1 diabetic with extensive, rapidly progressive macrovascular disease that is not associated with classical risk factors for CAD.

## Case presentation

**Figure 1:**
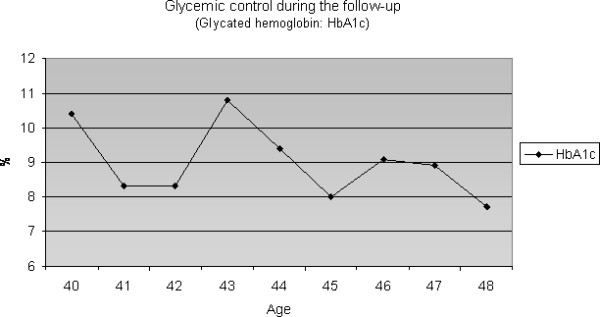
Glycemic control during the follow up.

This case study describes a 48-year-old female patient who was diagnosed with T1DM at the age of 16. The patient remained under regular follow-up while conducting a labile glycemic control by first using insulin NPH twice a day and, subsequently, using a long- acting human insulin analog (from 1999 to present) [Figure 1].

**Figure 2:**
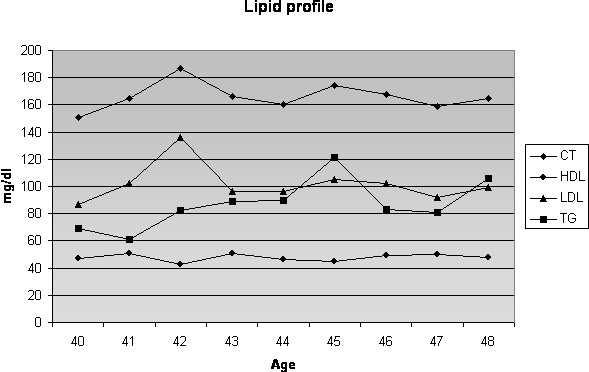
**Lipid profile**. Note that prior to coronary event at 42 years-old, she had a normal lipid profile.

**Figure 3:**
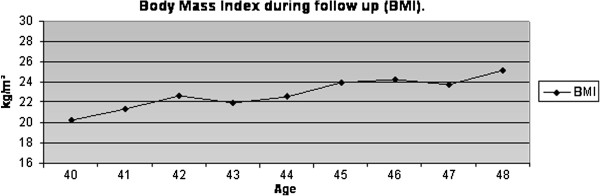
BMI during follow up.

**Figure 4:**
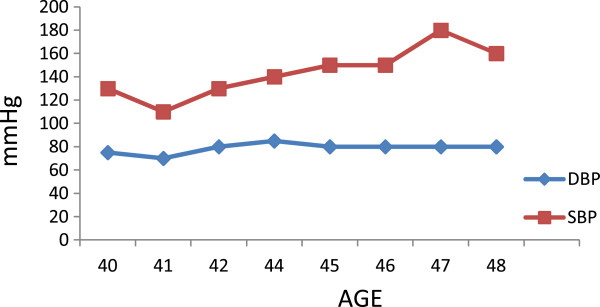
Blood pressure levels during follow up.

**Figure 5:**
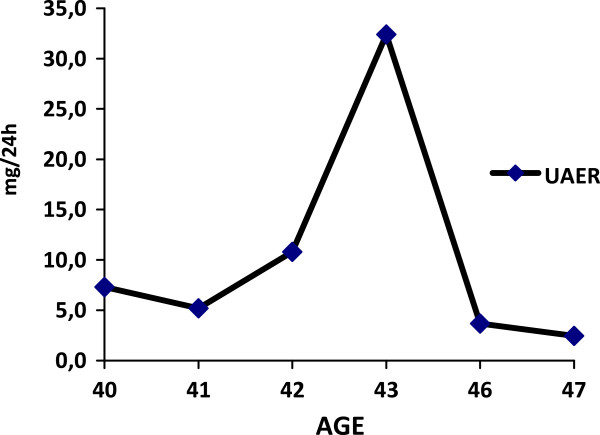
UAER levels during follow up.

There was no family history related to DM, systemic arterial hypertension or CAD. Smoking habits were absent during all her life. Her father died from stroke after the age of 70 years and her mother died from acute myocardial infarction when she was 75 years. Lipid profile and body mass index (BMI) and Blood pressure levels were normal, as were acute phase markers (C-reactive protein – CRP, homocysteine, apoprotein A and apoprotein B) [Figures 2, 3 and 4]. Accordingly, this patient had a low risk for CAD. Proteinuria levels were within normality until the age of 40 years and after this urinary albuminuria excretion rate (UAER) was either within normality [Figure 5].

**Figure 6:**
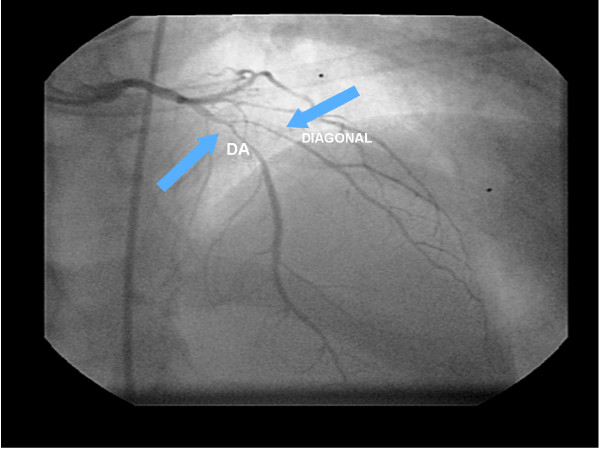
**The cineangiocornariography of the first acute myocardial infarction.** The left coronary artery disclosed extended stenosis of 90% at the Anterior Descending Artery (DA) after the first diagonal branch, and of 80% at the proximal segment of the diagonal artery.

**Figure 7:**
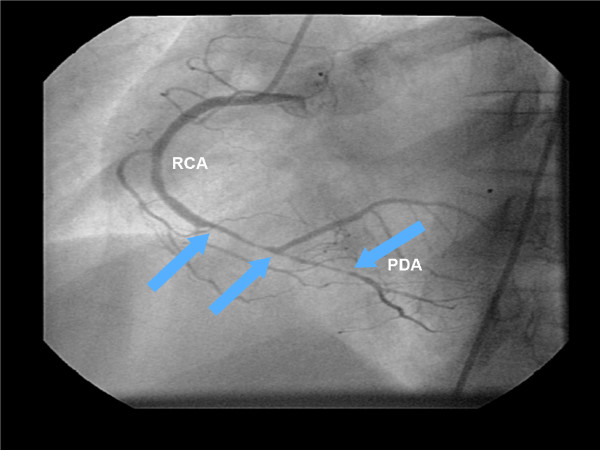
**The cineangiocornariography of the first acute myocardial infarction.** The right coronary artery presented an extended stenosis of 90% before its bifurcation and its posterior branches showed a stenosis of 90% at the medium segment. PDA: Posterior descending artery. RCA: right coronary artery.

**Figure 8:**
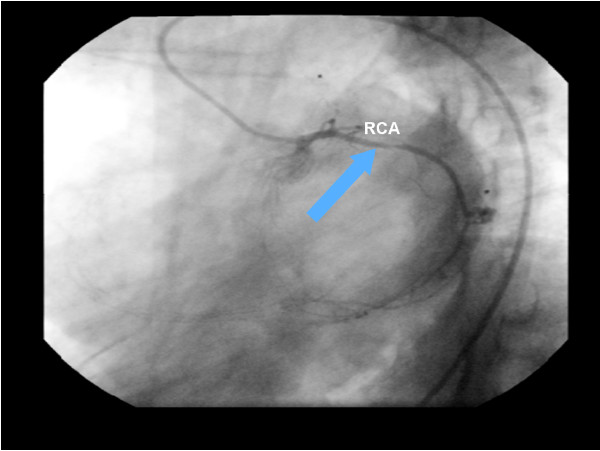
**The cineangiocornariography of the second acute myocardial infarction.** The Right Coronary Artery (RCA) in the proximal portion with obstruction of 80-90%.

At the age of 42 (26 years of disease evolution), she presented for the first time with clinical symptoms of acute coronary syndrome (ACS) with an acute myocardial infarction (AMI). The cineangiocoronarography (CAT) of the left coronary artery disclosed extended stenosis of 90% at the anterior descending artery (DA) after the first diagonal branch and 80% at the proximal segment of the diagonal artery. The right coronary artery presented an extended stenosis of 90% before its bifurcation, and its posterior branches showed a stenosis of 90% at the medium segment [Figures 6 and 7]. She was submitted to a myocardial revascularization by implanting an internal left mammary graft for DA, an internal right mammary graft for the diagonal artery, and an aorta coronary bypass for the marginal coronary artery. At the age of 44 (2 years after the first ACS event), she presented another AMI, this time involving the right coronary artery (RCA) in the proximal portion with obstruction of 80-90% [Figure 8]. Stents with rapamycin were immediately implanted at the right coronary artery. By this time, the echocardiogram disclosed no diastolic dysfunction, showing an ejection fraction of 70%.

At the age of 48, her last evaluation disclosed a predominantly systolic hypertension, and her myocardial scintigraphy showed an ischemia induced by effort, despite the optimized cardiovascular therapy.

## Discussion

This case report described a T1DM patient who did not present nephropathy, retinopathy or classical risk factors for CAD. However, during her chronic hyperglycemic evolution due to extremely difficult control (mean of HbA1c > 3 percentage points above the upper limit), she presented with early and aggressive coronary artery disease.

From a clinical point of view, it is well known that DM increases cardiovascular risk. During the last decades, several epidemiological studies demonstrated that some risk factors, including systemic arterial hypertension, diabetes, smoking, dyslipidemia [8], family history, sedentary lifestyle, central obesity and poor ingestion of fruits and vegetables, account for almost 90% of cases of CAD in the global population [4]. Nevertheless, while the precise reasons for this increase in risk remain unclear, kidney disease and the usual risk factors for CAD seem to contribute.

At present, the role of glycemic control as a contributive factor for CAD is still controversial. In recent decades, several clinical trials have investigated the effect of intensive treatment of hyperglycemia on cardiovascular risk reduction in T1D [9]. Although the complete role of hyperglycemia in the pathogenesis of cardiovascular diseases (CD) is still speculative, relevant studies, such as the The Diabetes Control and Complications Trial/Epidemiology of Diabetes Interventions and Complications (DCCT/EDIC) Study, have shown advantages to glycemic control through intensified insulin treatment for a reduction in macrovascular events. After a 17-year follow-up, it was shown that intensive diabetes treatment reduced the risk of any CD in 42% of patients and of non-lethal myocardial infarction and cerebrovascular accident in 57% of patients. This reduction in risk was mostly associated with the reduction in glycohemoglobin during DCCT [9].

The Pittsburgh Epidemiology of Diabetes Complications Study (EDC) found no significant relationship between fasting plasma glucose or exposure time to glucose and the incidence of CAD [10,11]. Another important study, the EURODIAB [12], corroborated the relevant predictor role of albuminuria in the pathogenesis of CAD in T1DM. It also verified a stronger link between macroangiopathy and metabolic syndrome markers than with hyperglycemia itself. Moreover, gender-specific risk for SAP (systolic arterial pressure), triglycerides (or HDL cholesterol), and the waist-to-hip ratio were associated with the progression of CAD. [12] The main lesson learned from these results is that intensive and early treatment of hyperglycemia in patients with short duration of diabetes and low cardiovascular risk leads to cardiovascular benefits.

Besides the classical risk factors and hyperglycemia, it is important to identify the specific risk predictors for CAD in T1DM. Nephropathy is a more significant risk predictor, which has already been recognized, confirmed and has long been associated with an overall increased mortality risk, particularly due to cardiovascular disease [13]. Besides reflecting the increase in insulin resistance, possibly the culprit in the pathogenesis of CAD in T1D, microalbuminuria is also a marker of impairment of renal function in its early stages as stated by Steno Investigators [14]. The endothelial dysfunction that usually precedes microalbuminuria [15] is probably the link between the development of renal and cardiovascular complications commonly seen in T1D. .Another complication usually involved in the risk for CAD is the cardiovascular autonomic neuropathy. In patients with long-term T1DM, the response of myocardial blood flow to sympathetic stimulation appears to be jeopardized inside the myocardial areas with autonomic denervation, indicating a defective vasodilator response of the coronary vessels. There are several mechanisms that may contribute to early death from CAD in these patients with autonomic neuropathy, including abnormalities in coronary vasomotor capacity, arrhythmia and changes in systolic and diastolic function, as the threshold is lower at the setting of a relative increase in sympathetic tone. Such situations are commonly seen in diabetic individuals with sympathovagal imbalance [6].

However, it is worth noting that there are many limitations to the existing risk predictor models. While some tools are available for the evaluation of risk of cardiovascular events in the population in general and for T2DM, these instruments were neither validated nor tested for T1DM. These models do not take into account a lower age range, nor do they consider several co-morbidities specifically related to DM. Therefore, they are likely to provide mistaken risk estimation for patients, including some that are high risk. Consequently, there is an obvious need for creating new specific risk predictor models for T1DM that encompass all of these factors [2].

The predisposition to unstable plaques in DM results from the abnormal metabolic conditions shown by these patients, which lead to alterations in endothelial, inflammatory and smooth muscle cells [16]. In addition, there is a strong tendency toward thrombosis due to functional alterations in platelets and within the coagulation cascade [17]. The metabolic abnormalities that characterize diabetes, such as hyperglycemia, increased free fatty acids and insulin resistance, trigger molecular mechanisms that contribute to vascular dysfunction and, consequently, to the increase in cardiovascular risk among T1DM patients [18,19] through both microvascular and macrovascular complications [20,21].

Genetics can also be important. Haptoglobin is considered as another antioxidant defense in diabetes, because this plasma protein binds free hemoglobin resulting in the inhibition of iron-induced oxidative damage. T1D patients with haptoglobin genotype 2-2, which determines lower antioxidant capacity, presented higher incidence of CAD after a follow-up of 18 years when compared to those with genotype 1-1 [22]. Moreover, the haptoglobin genotype 2-2 was found to confer a 2-fold increased risk of cardiovascular events (myocardial infarction, stroke and death), when compared to genotypes 1-1 and 2-1 in a recent meta analysis [23]. Furthermore, HLA class II genes that are expressed on infiltrated inflammatory cells and smooth-muscle cells in atherosclerotic plaques are independently associated with cardiovascular events and death in a Finnish T1D study.[24].

Gender is another relevant factor. Studies demonstrate that the impact produced by diabetes is higher among women than among men, yet the magnitude of these differences is still a matter of debate [25]. In an English cohort with more than 23.000 T1DM patients, CAD was responsible for 8% of deaths among men and 11% of deaths among women under 40 years old [26]. This study and other data also demonstrate the impact of T1DM on the mortality of young individuals [27], specifically female patients. The detection of CAD among women is crucial because 40% of all coronary events affecting female patients lead to death [28]. In addition, 67% of the sudden deaths of coronary origin are verified in women who presented no prior manifestation [29]. On the other hand, only 50% of those presenting suggestive symptoms of angina showed significant obstructive lesions after coronary angiography [30]. These data illustrate the fact that, among the female population, the diagnosis of CAD may be a great challenge. Interestingly, the influence of risk factors such as smoking, obesity and particularly diabetes is considerably higher among women than among men [31,32]. In addition, their post-AMI prognosis is also worse compared to males, with a higher mortality in the first year, reaching 38% among women in comparison with 25% among men [33]. An explanation of these phenomena may be that T1DM increases the prevalence and the severity of coronary calcification in women. The differences in gender associated with fat deposition and distribution of HDL and LDL cholesterol could explain why diabetes increases the coronary calcification and the frequency of CAD in women to a higher degree than in men [7].

## Conclusions

The rapidly progressive macrovascular disease observed in this case report, which focused a young type 1 diabetic patient without prior cardiovascular diseases, reflects the multiple phenotypes of diabetes, with different degrees of aggressiveness and unpredictable clinical evolution in some cases. Because diabetes undoubtedly leads to a higher risk of CD and a worse long-term prognosis, this case also confirms the need for multi-factorial, early and optimized clinical management through glycemic, pressure and lipid control, as well as the investigation and control of the specific co-morbidities.

## Consent

Written informed consent was obtained from the patient for publication of this case report. A copy of the written consent is available for review by the Editor-in-Chief of this journal.

## Abbreviations

ACS: Acute coronary syndromes; AMI: Acute myocardial infarction; CAD: Coronary artery disease; CAT: Cineangiocoronarography; CD: Cardiovascular diseases; DA: Descending artery; DM: Type 2 diabetes; RCA: Right coronary artery; SAP: Systolic arterial pressure; T1DM: Type 1 diabetes; UAER: Urinary albuminuria excretion rate.

## Competing interests

The authors declare that they have no competing interests.

## Authors’ contributions

ASMM participated in acquisition of data and drafting the manuscript. MBG participated in revising critically the manuscript and giving the final approval of the version to be published. All authors read and approved the final manuscript.
